# Digital Dilemmas: A Comprehensive Review of the Psychosocial and Sleep Effects of Web Streaming on the Indian Youth

**DOI:** 10.7759/cureus.51096

**Published:** 2023-12-26

**Authors:** Vaibhav Chandra, Abhay Gaidhane, Sonali G Choudhari, Zahiruddin Quazi Syed

**Affiliations:** 1 School of Community Medicine, Jawaharlal Nehru Medical College, Datta Meghe Institute of Higher Education & Research, Wardha, IND; 2 School of Epidemiology and Public Health, Jawaharlal Nehru Medical College, Datta Meghe Institute of Higher Education & Research, Wardha, IND

**Keywords:** cultural identity, digital consumption, sleep patterns, psychosocial effects, youth, web streaming

## Abstract

The rapid growth of web streaming platforms in India has ushered in a transformative era in entertainment consumption, particularly among the youth. This comprehensive review explores the psychosocial and sleep effects of web streaming on the younger generation. Examining the impact on social interactions, mental health, academic performance, cultural identity, and sleep patterns, the study delves into the intricate dynamics shaping the lives of Indian youth in the digital age. Key findings reveal the subtle yet significant changes in face-to-face interactions, the emergence of virtual relationships, and the potential influence on stress, anxiety, and depression due to addictive binge-watching behaviours. Moreover, the review highlights the challenges to academic performance through disruptions in study habits and increased screen time.

Cultural and identity influences, both in terms of representation and misrepresentation, are explored, emphasizing the need to analyze the portrayal of Indian culture in web content critically. The sleep effects of web streaming, including sleep deprivation due to late-night binge-watching and the consequences of blue light exposure on melatonin production, add a crucial dimension to the discussion. Technological solutions such as screen time limitations, parental controls, and educational programs promoting media literacy and responsible digital consumption emerge as potential coping mechanisms. In conclusion, the study provides a nuanced understanding of the complexities surrounding web streaming and its impact on the youth in India. The implications for policy and practice underscore the importance of a balanced approach to digital consumption. At the same time, a call to action emphasizes the need for collaborative efforts in promoting responsible digital habits among the youth.

## Introduction and background

In recent years, India has witnessed an unprecedented surge in the popularity and accessibility of web streaming platforms. The digital landscape, once dominated by traditional television, has undergone a profound transformation with the emergence of platforms offering a diverse array of content, ranging from movies and TV shows to user-generated videos. This shift is particularly pronounced among the younger demographic, signifying a substantial change in media consumption patterns [1]. The proliferation of affordable high-speed internet and the widespread availability of smartphones have fueled India's exponential growth of web streaming platforms. The market has become highly competitive, with local and international players vying for the attention of the Indian audience. This surge has redefined the entertainment landscape and ushered in a host of challenges, especially concerning the psychosocial well-being of the youth [2].

The youth in India, comprising a significant proportion of the population, have become ardent consumers of digital content. Web streaming services' convenience and personalized nature have contributed to a seismic shift in entertainment preferences. Understanding the profound implications of this shift is imperative, as it not only shapes the cultural and social experiences of the youth but also potentially impacts their mental health and well-being [3].

The primary objective of this review is to delve into the psychosocial impact of the widespread adoption of web streaming platforms among the youth in India. As these platforms become integral to daily life, exploring their influence on social interactions, mental health, and overall well-being is essential. By critically examining the evolving dynamics of digital entertainment consumption, we aim to shed light on the potential challenges and benefits that emerge in the wake of this technological revolution. In tandem with the psychosocial aspect, this review will investigate the often-neglected domain of sleep patterns among Indian youth in the context of web streaming. With the omnipresence of smartphones and the allure of binge-worthy content, a growing concern has surfaced regarding the impact of late-night streaming on sleep quality and duration. By examining existing research and identifying gaps in our understanding, this review seeks to provide a comprehensive picture of the intricate relationship between web streaming habits and sleep hygiene among the youth in India.

## Review

Prevalence

Indian youth spend an average of eight hours and 29 minutes watching online video content, which is more than the global trend of six hours and 45 minutes [4]. The study found that 54% of the respondents were male, while 46% were female, indicating that females are equally interested in web streaming content [4]. The most popular online platforms for streaming video content among Indian youth include Netflix, YouTube, Hotstar, and Sony LIV [4]. Web streaming content can have various psychological effects on the Indian youth, such as enhancing criminal activity, changing language and behavior, and affecting physical exercises [4]. For example, 40.2% of respondents felt that web series enhance criminal activity in society, while 62.4% agreed that web series change the language and behavior of the youth [4]. A study on the inclination of Indian youth towards video streaming platforms identified factors such as convenience, accessibility, and the availability of diverse content as reasons for their preference for these platforms over traditional television shows [5]. These findings suggest that web streaming content has become increasingly prevalent among the Indian youth, with various psychological and behavioral effects. However, more research is needed to understand the psychosocial and sleep effects of web streaming on Indian youth comprehensively.

Psychosocial effects of web streaming

Social Isolation

Impact on face-to-face interactions: The widespread adoption of web streaming platforms has become a focal point of concern regarding its impact on traditional face-to-face interactions, particularly within the youth demographic. The prevailing culture of binge-watching, characterized by individuals immersing themselves in prolonged viewing sessions, can potentially relegate traditional social gatherings and real-world engagements to a secondary role. As the allure of on-demand content grows, there is a subtle yet perceptible decline in direct interpersonal connections. The shift towards solitary and screen-mediated entertainment raises questions about the overall health of face-to-face interactions in the digital age. This transformation in social behaviour prompts reflection on how the digital landscape reshapes the dynamics of personal connections and the potential consequences for the social fabric [4].

Virtual relationships and their implications: Contrary to the potential decline in face-to-face interactions, the rise of web streaming has given birth to a distinctive form of social interaction, the virtual relationship. The shared experience of consuming series or movies online has led to online communities and forums where individuals connect based on shared interests and preferences. While fostering a sense of belonging within these virtual communities, this digital camaraderie prompts critical questions about the authenticity and depth of relationships formed without physical presence. The nature of these virtual relationships, thriving on shared interests rather than physical proximity, challenges conventional notions of social connectedness. This phenomenon raises intriguing implications for the evolving dynamics of social bonds in the digital era, prompting a reevaluation of what constitutes meaningful social interaction in a landscape dominated by screen-mediated connections [6].

Mental Health

Addiction and binge-watching behaviours: The contemporary landscape of web streaming platforms has introduced a phenomenon marked by the allure of continuous streaming, often exacerbated by autoplay features. This has given rise to a notable increase in addiction and binge-watching behaviours among users. The seamless access to an extensive library of content, available at any time of the day or night, fosters a compulsive need to consume, leading to prolonged viewing sessions [7]. This behavioural pattern can disrupt daily routines, compromise work obligations, and adversely affect healthy lifestyle habits. The accessibility and abundance of content and autoplay functionalities contribute to a compulsive and potentially detrimental engagement with web streaming platforms [8].

Influence on stress, anxiety, and depression: The intersection between excessive web streaming and mental health concerns has become a focal point of investigation. The immersive nature of content consumption, combined with the emotional intensity of specific narratives, has been identified as a potential contributor to heightened stress, anxiety, and symptoms of depression [9]. While digital content may temporarily help escape reality, its prolonged use as a coping mechanism raises critical questions about its impact on long-term psychological well-being. Studying this relationship is essential for understanding the intricate interplay between digital entertainment and mental health, shedding light on potential risks and implications for individuals who heavily rely on web streaming as a means of emotional regulation [10].

Influence on academic performance

Time Management Challenges

Impact on study habits and academic commitments: The advent of on-demand content through web streaming platforms has introduced a noteworthy challenge to traditional study habits, particularly among students. The accessibility and allure of continuous, uninterrupted viewing create a temptation that potentially disrupts the allocation of dedicated time for academic pursuits. The convenience of accessing a diverse range of content at any moment may entice students into indulging in binge-watching sessions, leading to procrastination and compromising the quality of their study sessions. This shift in study behaviour highlights the importance of understanding how web streaming habits impact academic commitments, emphasizing the need for a nuanced approach to address the potential challenges that arise [11-12]. Recognizing this impact becomes crucial for ensuring that students maintain a healthy balance between academic responsibilities and digital entertainment.

Relationship between screen time and academic performance: The evolving relationship between screen time and academic performance is a pivotal aspect of students' digital dilemma. The increased screen duration, whether for educational or entertainment purposes, has potential implications for cognitive functions and attention spans [13]. As students navigate a digital landscape encompassing academic and leisure screen time, questions surface regarding the direct correlation between heightened screen time, possibly fueled by web streaming habits, and a subsequent decline in academic achievement. Understanding this intricate relationship becomes essential for educators, parents, and policymakers alike. Developing strategies that foster a balanced approach to screen usage is imperative for safeguarding students' academic success and overall well-being in the digital age [14]. This acknowledgement sets the stage for informed interventions prioritising students' educational pursuits while navigating the digital landscape.

Cultural and identity influences

Portrayal of Indian Culture in Web Content

Representation and misrepresentation: The content disseminated by web streaming platforms is pivotal in shaping perceptions of Indian culture, contributing significantly to constructing a virtual narrative. These platforms showcase various cultural elements, traditions, and lifestyles, potentially offering a nuanced portrayal of the richness and diversity of Indian culture. However, concerns arise when this representation devolves into stereotypes or misrepresentations, diminishing the authenticity of cultural portrayals. When cultural depictions are reduced to clichés or inaccuracies, it perpetuates narrow views that fail to capture the multifaceted nature of Indian society authentically. The risk of misrepresentation within the virtual space underscores the responsibility of platforms to curate content responsibly, ensuring it respects and accurately reflects the rich tapestry of Indian culture [15]. This recognition prompts a call for content creators and platforms to engage in thoughtful and culturally sensitive storytelling, contributing to a more accurate and inclusive representation of Indian identity.

Influence on cultural perceptions and values: The narratives presented through web streaming platforms wield significant influence over cultural perceptions and values, especially among the youth. Whether authentic or skewed, these narratives contribute to shaping the cultural lens through which the audience views their identity and that of others. This influence's subtle yet profound impact raises questions about its potential ramifications on cultural values and the reinforcement or challenge of societal norms. In an era where the virtual world increasingly serves as a primary source of cultural information, understanding the dynamics of this influence becomes paramount. It prompts reflection on the responsibility of content creators and platforms to contribute positively to the cultural landscape. Fostering an appreciation for diversity and facilitating a nuanced understanding of Indian identity and values are integral aspects of this responsibility [16-17]. This acknowledgement underscores the transformative potential of media in influencing cultural narratives. It emphasizes the need for a conscientious approach to content creation that aligns with the diverse and dynamic reality of Indian society.

Sleep effects of web streaming

Sleep Deprivation

Late-night binge-watching habits: The accessibility and convenience provided by web streaming platforms have given rise to a prevalent and potentially concerning phenomenon: late-night binge-watching. The allure of continuous, on-demand content tempts viewers to engage in just one more episode or film, leading to extended screen time well into the night. This behaviour, if habitual, poses a substantial risk of sleep deprivation, blurring the boundary between leisure and rest. The disruption of traditional sleep schedules due to late-night binge-watching raises concerns about its impact on overall sleep hygiene and subsequent effects on physical and mental well-being. The evolving trend of late-night binge-watching necessitates a deeper understanding of its implications for individuals' sleep health and the broader consequences on their overall quality of life [18].

Impact on overall sleep quality and duration: The cumulative effect of late-night binge-watching on sleep quality and duration is emerging as a pressing concern in the digital age. Sleep, a process driven by quantity and quality, is integral to maintaining physical and mental health. The intrusion of web streaming habits into nightly routines can compromise the restorative nature of sleep. Prolonged screen exposure during late-night viewing sessions may disrupt the natural sleep-wake cycle, impacting cognitive functions, mood, and overall health. Recognizing the interconnectedness of web streaming habits and sleep quality becomes crucial for understanding the potential risks of this behaviour. Promoting healthier sleep patterns among individuals who engage in late-night binge-watching involves addressing the broader implications on well-being and fostering awareness of the importance of sleep hygiene in the digital era [19].

Blue Light Exposure

Influence on melatonin production: The screens utilized for web streaming emit blue light, a specific wavelength recognized for suppressing melatonin production, the hormone responsible for regulating sleep-wake cycles. Exposure to blue light, especially during the evening hours, can disrupt the natural circadian rhythm by inhibiting melatonin secretion. This interference with melatonin levels may lead to difficulties falling asleep, as the body's internal signals for sleep initiation are compromised. The prevalence of prolonged exposure to blue light from screens, a common occurrence during web streaming sessions, underscores the importance of understanding how such exposure can potentially decrease sleep efficiency and disrupt the overall quality of sleep [20]. Recognizing the impact of blue light on melatonin production becomes a critical consideration for individuals navigating the digital landscape, particularly during nighttime screen activities.

Consequences for circadian rhythm and sleep-wake cycle: The consequences of blue light exposure extend beyond melatonin suppression, influencing the broader circadian rhythm, the body's internal clock regulating various physiological processes. Disruption of the natural sleep-wake cycle becomes a significant outcome, manifesting in irregular sleep patterns, daytime sleepiness, and an increased vulnerability to sleep disorders. The ubiquity of web streaming as a nighttime activity intensifies exposure to blue light during a critical period for the body's preparation for sleep. Understanding and mitigating the impact of blue light on the sleep-wake cycle are imperative for maintaining healthy sleep hygiene. Efforts to implement technologies or habits that reduce blue light exposure during nighttime web streaming sessions play a vital role in safeguarding the integrity of individuals' sleep-wake cycles, contributing to overall well-being and sleep quality [21].

Coping mechanisms and adaptations

Technological Solutions

Development of features to limit screen time: In response to the recognized potential for excessive screen time and its associated impacts, various web streaming platforms have taken proactive measures by introducing features designed to limit and manage viewing durations. These tools empower users to set time restrictions on their viewing sessions, receive notifications signalling prolonged usage, and even schedule breaks to interrupt continuous streaming. The overarching goal is to cultivate a conscious and intentional approach to content consumption, thereby preventing the unintended consequences of overindulgence. By providing users with these features, web streaming platforms contribute to promoting a balanced and mindful interaction with digital content. This acknowledgement of the impact of prolonged screen time on well-being reflects a commitment to user health. It underscores the platforms' role in fostering responsible digital habits among their audience [22].

Parental controls and their effectiveness: The integration of parental control features within web streaming platforms functions as a critical instrument for guardians seeking to monitor and regulate the content consumption of younger users. These controls typically offer functionalities such as setting age-appropriate content filters, imposing restrictions on screen time, and tracking the viewing history of the younger audience. However, the effectiveness of these controls hinges on their usability and the active involvement of parents in overseeing and managing their children's digital activities. The willingness of parents to engage with these tools, coupled with the platforms' commitment to user-friendly design, determines the efficacy of parental controls in creating a safer and age-appropriate digital environment for younger viewers. The successful implementation and utilization of parental controls underscore the collaborative responsibility shared by both parents and platforms in ensuring a secure and healthy digital experience for children [23].

Educational Programs

Media literacy initiatives: Media literacy initiatives are pivotal in empowering individuals, particularly the youth, with essential skills to critically analyze and navigate the expansive content landscape on web streaming platforms. Educational programs within this domain focus on enhancing digital literacy, providing users with the tools to discern between authentic and misleading information. By fostering a thoughtful approach to media consumption, these initiatives contribute to developing a discerning audience capable of navigating the diverse content offered by web streaming platforms. The emphasis is on arming users with the skills necessary to make informed choices, promoting a more nuanced understanding of the digital content landscape. In a rapidly evolving digital era, media literacy initiatives serve as a cornerstone for cultivating a digitally literate population that can confidently navigate the complexities of online content [24].

Promotion of responsible digital consumption: Moving beyond literacy initiatives, there is a pressing need to promote responsible digital consumption actively. Educational campaigns emphasize the importance of balance in engaging with web streaming content, encouraging users to integrate digital entertainment into their lives in a manner that complements rather than disrupts daily routines. These campaigns highlight the potential psychosocial and health implications of excessive digital use, fostering a culture of mindful engagement. By raising awareness about the impact of web streaming habits on well-being, these campaigns aim to instil a sense of responsibility among users, encouraging them to adopt healthier and more balanced digital consumption patterns. The focus is on empowering users to make conscious choices about their digital engagement, promoting a culture of responsible and mindful use of web streaming platforms in the digital age [25].

Recommendations for future research

Longitudinal Studies on the Long-Term Psychosocial Effects

Given the dynamic nature of digital culture and the pervasive influence of web streaming on the youth, the imperative for comprehensive longitudinal studies becomes evident. These studies aim to track the long-term psychosocial effects of web streaming over an extended period, delving into changes in social behaviour, mental health outcomes, and overall well-being. Researchers can garner valuable insights into the enduring implications of sustained digital content consumption [4].

Longitudinal studies provide a unique vantage point, allowing researchers to observe and analyze how patterns of web streaming engagement evolve and their consequential effects on various aspects of the youth's lives. This approach is crucial for discerning whether the initial trends observed in the short term persist, intensify, or diminish over months or years [26]. The insights gleaned from such longitudinal studies can inform policymakers, educators, and healthcare professionals about the nuanced and potentially evolving impacts of web streaming on the psychosocial well-being of the youth.

This knowledge is essential for crafting informed interventions, educational strategies, and mental health support systems tailored to the evolving needs of the digital generation [27]. As digital behaviours continue to evolve, longitudinal studies serve as an invaluable tool for staying ahead of emerging trends and understanding the long-term consequences of the digital revolution on the youth's well-being.

Investigation into the Effectiveness of Interventions

As technological solutions and educational programs are developed and implemented to address the challenges posed by web streaming, there is a crucial need for rigorous evaluation of their effectiveness. Future research should focus on assessing the impact of interventions, including but not limited to screen time limitations, parental controls, and media literacy initiatives [28]. An in-depth investigation into the effectiveness of these interventions is essential for several reasons. Firstly, it provides empirical evidence on whether these strategies successfully achieve their intended goals, reducing excessive screen time, enhancing parental oversight, or fostering a more discerning and responsible digital audience. Secondly, understanding the nuances of how these interventions operate in diverse contexts and among different demographics ensures that strategies can be tailored to specific needs and challenges [29].

Research in this domain is pivotal for informing policy and practice. Policymakers can use evidence-based insights to shape regulations and guidelines that effectively safeguard the well-being of users, especially the youth. Educators can adapt and refine educational programs based on proven outcomes, while technology developers can fine-tune features to enhance user experience and support healthy digital habits [30]. Investigating the effectiveness of interventions is a dynamic process that contributes to developing strategies to mitigate adverse outcomes associated with web streaming and promote a culture of responsible digital engagement. As the digital landscape continues to evolve, ongoing research and evaluation will be essential for ensuring that interventions remain relevant and effective in addressing the challenges posed by the growing influence of web streaming on users' well-being.

Exploration of Cultural Nuances in the Impact of Web Streaming

The diverse cultural landscape of India adds a layer of complexity to the influence of web streaming on the youth, necessitating comprehensive research to explore the cultural nuances that shape the reception and interpretation of digital content within the Indian context [31]. This exploration involves a multifaceted investigation into how regional, linguistic, and socio-economic factors influence how the youth engage with and are influenced by web streaming platforms. Cultural nuances play a pivotal role in determining digital content preferences, viewing habits, and interpretations. For example, the portrayal of cultural elements in content may resonate differently with audiences from various regions, linguistic backgrounds, and socio-economic strata [32].

Understanding these nuances is crucial for several reasons. Firstly, it allows for the development of content that is more inclusive, respectful, and reflective of the diverse cultural identities within India. By acknowledging and embracing the richness of India's cultural tapestry, content creators can create narratives that resonate authentically with a broad audience. Additionally, this understanding provides insights into how digital content may impact cultural perceptions, values, and identity formation among the youth [33]. Research in this domain contributes to a more nuanced understanding of the intersection between digital media and culture, acknowledging the dynamic and context-specific nature of the impact of web streaming on the diverse youth population in India. This nuanced understanding can inform content creators, platforms, and policymakers to tailor culturally sensitive strategies that resonate positively with the diverse audiences in the country. By recognizing and embracing cultural diversity, the digital content landscape can contribute to fostering a sense of inclusivity and cultural appreciation, enhancing the overall digital experience for India's youth.

## Conclusions

In conclusion, examining web streaming's impact on the psychosocial well-being and sleep health of the youth in India reveals a complex interplay between digital consumption and various facets of life. The findings underscore the need for a nuanced understanding of the social, mental, and cultural dimensions influenced by web streaming platforms. Policymakers and educators must take heed of these insights, shaping guidelines and educational initiatives that foster responsible digital habits. Media literacy programs integrated into curricula, accessible parental controls, and features limiting screen time emerge as crucial tools. There is a pressing call to action for promoting responsible digital habits among Indian youth, emphasizing a balance between the benefits of digital content and the potential risks associated with excessive consumption. Collaborative efforts are necessary from parents, educators, policymakers, and technology developers to create an environment that supports the well-being of the youth in the digital age, ensuring a future where technology complements, rather than compromises, their overall health and development.
